# Research on the Effects of Extruded Okara on Improving Gluten Protein Stability and Dough Processing Properties

**DOI:** 10.1002/fsn3.70656

**Published:** 2025-07-15

**Authors:** Fengchen Zhou, Yangyang Cui, Shiming Wang, Xianjun Zou, Zhilong Chen, Yakun Shan, Yu Zhang, Sheng Li

**Affiliations:** ^1^ College of Food Science and Engineering Changchun University Changchun China

**Keywords:** dietary fiber, dough rheology, okara, protein, single‐screw extrusion

## Abstract

Okara is a byproduct produced after grinding soybeans, but its poor taste and difficulty in storage have posed a severe challenge in handling it. This study aims to modify okara using single‐screw extrusion technology to alter the ratio of soluble dietary fiber (SDF) to insoluble dietary fiber (IDF) and to investigate their effects on the protein conformation, rheological properties, and microstructure of wheat dough. The experimental results indicate that the SDF content in extruded okara was significantly increased by approximately 6.39%, with the swelling ability and hydration capacity increasing by 1.69 (g/g) and 2.4 (g/mL), respectively. The addition of 6%–9% extruded okara into the dough caused a redistribution of water, increased the proportion of bound water, altered the protein conformation, and increased the content of the α‐helix structure. At the 9% addition level, compared to the control group, the content of α‐helix increased by 4.45%, leading to the conversion of β‐turns to β‐sheets and forming a more stable gluten network structure. However, compared to untreated raw okara, adding extruded okara increased the dough's elastic modulus and loss modulus, lowered the tanδ value, reduced the pasting viscosity, and improved the viscoelastic properties of the dough. These findings deepen our understanding of the mechanism by which extruded, modified okara added to wheat dough affects its properties and provide theoretical guidance for applying extrusion modification technology in okara processing and the development of bakery products.

## Graphical abstract

The okara was modified by single‐screw extrusion to convert soluble dietary fiber into insoluble dietary fiber, and then the modified okara was added to the dough to investigate the changes in the gluten protein network structure.

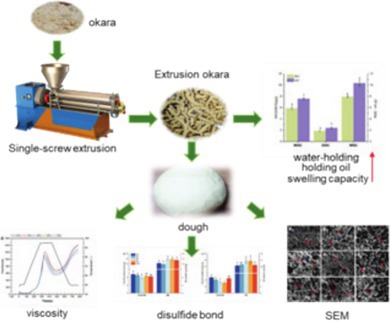

## 1 Introduction

With the increasing demand of people for healthy foods and low‐calorie diets, the development of products rich in dietary fiber (DF) has become a current hot topic in the field of food research (Zhang et al. 2022). Okara is the residue left after grinding soybeans for the production of tofu or soymilk, and for every kilogram of soybean processed for tofu, 1.2 kg of fresh okara can be produced (Feng et al. 2021). The increasing processing of soybeans worldwide can generate millions of tons of okara annually (Tuly and Ma 2024). Okara is a nutrient‐rich byproduct containing protein, fat, and DF. Among them, DF accounts for more than half of the dry weight, making okara an ideal source of DF (Li et al. 2025). DF can be divided into SDF and IDF according to its solubility in water. Among them, IDF accounts for more than 90% of the total DF content. Existing research has shown that SDF has more extensive physiological functions than IDF, such as lowering blood pressure, regulating blood sugar, and preventing colon cancer, among other health‐care effects (Ullah et al. 2018). However, the current utilization of okara is not satisfactory. Usually, it is only used as animal feed or treated as waste. This is mainly because the content of SDF in okara is low, while the content of IDF is high, resulting in poor taste and rough texture. If it is added to food products, it will reduce the quality of the products and make them lose the opportunity for value‐added (Hu et al. 2022). Therefore, it is necessary to modify okara to improve its utilization rate.

Okara, as a raw material with high DF content, has been widely used in high‐dietary‐fiber pasta. Research has indicated that in the dough system, the effects of DF on the rheological properties of the dough are complex and reciprocal (Liu et al. 2019). On the one hand, the high water‐holding capacity of okara DF favors the formation of the gluten network structure, increasing the dough's elasticity and flexibility. The interaction between moderate amounts of DF and gluten proteins leads to the formation of complexes, which contribute to the formation of a cohesive and stable gluten network structure, thus improving the properties of high DF products. On the other hand, the dilution effect of DF and the competition for water uptake could disrupt the gluten network structure. Excessive interaction between DF and gluten proteins hinders the cross‐linking between gluten proteins and starch, disrupting the gluten network structure. This can lead to deterioration of the rheological properties of the dough and, ultimately, product quality (Wu et al. 2025). Therefore, it is essential to understand the effect of DF on the rheological properties of the dough and to find corresponding countermeasures to improve it, not only for the high‐value utilization of okara to enhance nutritional value but also to solve problems of rough texture, poor taste, and poor product quality. There are many ways to modify PDFs. With the reduction of chemicals used in food production, more researchers and scholars are using physical modification technology to achieve better functionality. Physical processing techniques such as screw extrusion, steam explosion, ultra‐high pressure, and microwave treatment have been shown to change the physicochemical properties of DF and alter its composition. For example, Ouyang et al. (2023) modified DF in pomelo using ultra‐high pressure, which resulted in a significant increase in SDF from 2.49% ± 0.23% to 41.92% ± 0.32% and enhanced water‐ and oil‐holding capacity as well as swelling capacity. Zhao et al. (2024) modified okara using steam blasting to increase SDF content by up to 30.31%, which is 4.86 times higher than that of untreated okara SDF.

Extrusion processing technology has been widely used in food processing, becoming a primary means of processing DF, with solid continuity, high yield, and other favorable characteristics (Chien et al. 2022). When the material moves in the cavity, because of the high temperature, high pressure, and shear force generated by the extrusion equipment, rapid vaporization of water occurs, and the intermolecular and intramolecular spatial structure of macromolecular polymers expands and deforms. At the moment of extrusion, the die mouth produces dramatic structural changes due to the loss of pressure, forming a loose and porous state. Higher shear force can cut the glycosidic chains of cellulose and hemicellulose and reduce their molecular weights to form SDF. In addition, the extrusion process can cause changes in the raw material components, including protein denaturation, starch pasting, reduction of microbial content, and inactivation of antinutritional factors (Ma et al. 2023). Using extruded rice bran, Qiao et al. (2021) found an increase in SDF water‐holding, oil‐holding, and redox capacity. Li, Hu, et al. (2022) and Li, Zhou, et al. (2022) used twin‐screw extrusion to modify corn bran, increasing the SDF content from 2.42% to 6.54%. Modifying SDF content through extrusion is a hot topic in DF research. It can be seen that extrusion technology, as a physical modification method, has achieved certain results in the processing of various raw materials rich in DF.

Although there have been numerous studies on DF modification and okara utilization, most existing research focuses solely on enhancing the SDF content in okara through modification techniques. There is a lack of in‐depth analysis of the changes in the interaction between modified okara and gluten proteins when added to dough. In this study, okara was used as the raw material, and the extrusion method was employed to alter the ratio of SDF to IDF in okara. The changes in the interaction between different types of okara and gluten proteins in the dough system were systematically revealed. By adding okara with different ratios before and after extrusion to high‐gluten wheat flour, a comprehensive study was conducted on its effects on the farinographic properties, pasting properties, gluten proteins, and rheological properties of the wheat flour blend. The impact of okara before and after extrusion on the microstructure of wheat dough was observed, and the influence rules of different addition amounts of okara on the properties of wheat dough were explored. This study provides a new approach for the later development of okara as a raw material for food processing.

## 2 Materials and Methods

### 2.1 Materials

The okara was supplied by Yuxiang Bean Products Co. Ltd. in Changchun City, Jilin Province. The wheat flour was purchased from Xinliang Bread Flour in Xinxiang, Henan Province; the single‐screw extruder was purchased from Changchun Intelligent Instrument and Equipment Co. Ltd. (Model: ZJL—200B); the high‐speed pulverizer was purchased from Wuhan Haina Electric Appliance Co. Ltd. (Model: ZG—0313).

### 2.2 Experimental Methods

#### 2.2.1 Preparation of Okara Samples

Okara was dried at 55°C for 24 h and pulverized to 80 mesh sieve using a high‐speed pulverizer to obtain Raw Okara (RO). Then, a single‐screw extruder with a length‐to‐diameter ratio of 25 was used, and preliminary experiments were conducted to determine the optimal processing conditions. It was finally determined that the temperatures of the five heating cylinders of the machine were set to 80°C, 100°C, 120°C, 140°C, and 160°C, and the rotational speed of the screw was set to 120 r/min. The moisture content of the okara was 38%. These are the optimal processing conditions. After extrusion, the okara was dried in an oven at 55°C, then crushed using a high‐peed pulverizer and sieved through an 80‐mesh sieve for later use to obtain Extruded Okara (EO).

#### 2.2.2 Determination of the Elemental Composition of Okara

According to the method described by Agu et al. (2023), moisture, DF, SDF, IDF, protein, fat, and ash content in okara were determined.

#### 2.2.3 Measurement of Hydration Capacity Before and After Extrusion of Okara

Following the method by Wang et al. (2022), with slight modifications, water‐holding capacity (WHC) and oil‐holding capacity (OHC) were determined. One gram of sample was mixed with 30 mL of distilled water or soybean oil, placed at 4°C for 24 h, and then centrifuged at 6000 rpm for 10 min. After discarding the supernatant, the sediment was weighed. WHC is calculated based on Equation (1), while OHC is determined using Equation (2):

(1) $$\mathrm{WHC}\;\left(g/g\right)=\left(W_{2}-W_{1}\right)/m\times 100\%$$

In the formula, (*W*_1_) represents the weight (g) of the dried sample and centrifuge tube, (*W*_2_) represents the weight (g) of the centrifuge tube after sample absorption, and m represents the final dry weight (g) of the sample.

(2) $$\mathrm{OHC}\;\left(g/g\right)=\left(O_{2}-O_{1}\right)/m\times 100\%$$

In the formula, (*O*_1_) represents the weight (g) of the dried sample and centrifuge tube, (*O*_2_) represents the weight (g) of the centrifuge tube after sample absorption, and m represents the final dry weight (g) of the sample.

Water swelling capacity (WSC) was determined according to the method by Wang et al. (2021). A sample of 0.5 g was taken in a 15 mL stoppered test tube, to which 10 mL of distilled water was added. The mixture was gently stirred to remove air bubbles. After hydration at 25°C for 24 h, the volume change before and after was recorded. WSC is calculated according to Equation (3):

(3) $$\mathrm{WSC}\;\left(g/\mathrm{mL}\right)=\left(V_{1}-V_{0}\right)/m\times 100\%$$

where, (*V*_1_) is the volume after swelling, (*V*_0_) is the volume before swelling, and (m) is the weight of the dry sample.

#### 2.2.4 Preparation of Blended Wheat Flour

Before the experiment, RO and EO were mixed with wheat flour in different proportions (0%, 3%, 6%, 9%, and 12%). The mixtures were placed in self‐sealing bags filled with air, followed by vigorous shaking to ensure thorough mixing. Finally, the mixtures were stored at 4°C for later use.

#### 2.2.5 Determination of Pasting Characteristics of Wheat Flour

Regarding the method with slight modifications by Li, Wang, Jiang, et al. (2023) and Li, Wang, Wang, et al. (2023), a mixture of wheat flour (3.0 g, 14% wet basis moisture correction) with varying levels of added okara was prepared. This mixture was then combined with distilled water (25 mL) for viscosity measurement using a Rapid Visco Analyzer (RVA). The testing conditions were as follows: held at 50°C for 1 min, with the rotational speed starting at 960 rpm during this stage; after 10 s, the speed was reduced to 160 rpm, heated to 95°C within 3.75 min; held at 95°C for 2.5 min, then cooled to 50°C within 3.75 min, and finally held at 50°C for 2 min to obtain the viscosity curve.

#### 2.2.6 Determination of Flour Quality Characteristics of Wheat Flour

The experiment used a Farinograph (Brabender, Germany) with a 50 g mixing bowl. The effect of different levels of RO and EO additives (0%–12%) on the flour properties during the dough mixing process was investigated. Flour properties were characterized by parameters such as water absorption (WA, %), development time (DT, min), stability time (ST, min), and weakening degree (Ds, FU).

#### 2.2.7 The Determination of Free Sulfhydryl (SH_F_ )groups and Disulfide Bond (SS)in Wheat Dough

Referencing the method with slight modifications by Yang et al. (2017), the dough was kneaded using a farinograph until formation, then immediately removed and freeze‐dried. The freeze‐dried dough was then sieved through a 100‐mesh screen, and 0.075 g was weighed and placed into a 10 mL centrifuge tube. To this, 1 mL of buffer solution and 4.7 g of guanidine hydrochloride were added, and the volume was adjusted to 10 mL with buffer solution. For determination of SHF content, 1 mL of the sample solution, 4 mL of urea–guanidine hydrochloride solution, and 0.04 mL of DTNB reagent (4 mg/mL) were mixed in a 10 mL test tube. The absorbance was then measured at 412 nm using a UV spectrophotometer. Each sample was measured in triplicate, and the average was taken. The SH_F_ content was calculated according to Equation (4).

(4) $${\mathrm{SH}}_{F}\;\left(\mu \;\mathrm{mol}/g\right)=73.53\times A_{412}\times D/C$$

In the equation, 73.53 represents the molar absorption coefficient of DTNB, *A* 412 is the absorbance of the solution at 412 nm, *D* is the dilution factor, and *C* is the sample concentration in mg/mL.

To determine total sulfhydryl (SH) content, 1 mL of sample solution was mixed with 0.05 mL of β‐mercaptoethanol and 4 mL of urea–guanidine hydrochloride solution and stored at 25°C for 1 h. Subsequently, 10 mL of 12% trichloroacetic acid (TCA) was added to the mixture and kept at 25°C for another hour, followed by centrifugation at 5000 rpm for 10 min. The residue was washed twice with 5 mL of 12% TCA, then dissolved in 10 mL of 8 mol/L urea. Then, 0.05 mL of Ellman's reagent was added to the resulting solution, and the absorbance at 412 nm was measured. Each sample was measured in triplicate, and the average was taken. The SS bond content was calculated using Equation (5).

(5) $$\mathrm{SS}\;\left(\mu \;\mathrm{mol}/g\right)=\left(\mathrm{SH}–{\mathrm{SH}}_{F}\right)/2$$

In the equation, “SS” denotes the SS content expressed in micromoles per gram (μ mol/g).

#### 2.2.8 Wheat Dough Fourier Transform Infrared Spectroscopy (FTIR) Analysis

From Section 2.2.7, freeze‐dried dough powder was obtained and mixed with KBr (1:100, w/w) according to the method described by Bai et al. (2024). Subsequently, the mixture was ground into a fine powder using an agate mortar and pestle for pellet pressing. FTIR (ALPHA, Bruker, USA), full‐range scanning was conducted, with air as the control group. FTIR spectra within the 400–4000 cm^−1^ range were recorded at a resolution of 4 cm^−1^, accumulating 32 scans.

Processing of FTIR: Omnic software and Peak Fit 4.12 software were employed for comprehensive data analysis, focusing on studying the amide I band. Each experiment was conducted three times to ensure accuracy and consistency.

#### 2.2.9 Determination of the Texture of Wheat Doughs

According to the method with slight modifications by Li, Hu, et al. (2022) and Li, Zhou, et al. (2022), the dough was kneaded using a Farinograph until the development time, then immediately removed and allowed to rest for 30 min at 20°C. The dough was then molded into cubes with dimensions of 50 mm in length and 25 mm in height using homemade molds, resulting in Raw Okara Dough (ROD) and Extruded Okara Dough (EOD). A texture analyzer (SMS, UK) equipped with a P/36R probe was used for testing with the following settings: testing mode: compression; test type: TPA (Texture Profile Analysis); compression rate: 50.0%; pre‐, mid‐, and post‐test speeds: 3, 1, and 3 mm/s, respectively; trigger type: Auto; trigger force: 5 g; interval between compressions: 5.0 s.

#### 2.2.10 Determination of Dynamic Rheological Properties of Wheat Doughs

According to the method described by Gu et al. (2022), the dough was first kneaded using a Farinograph until development and immediately removed afterward. Dough samples with varying additive levels were allowed to rest at 25°C for 30 min after extrusion. Subsequently, a portion of the dough from the center was taken and spread evenly on the test platform of a rheometer (Brookfield, USA), and the excess dough was trimmed with scissors. To prevent water loss, the edges of the dough were coated with silicone oil. Dynamic rheological tests were then conducted using oscillatory mode under the following conditions: plate diameter of 40 mm, gap distance of 1 mm, temperature at 25°C, strain of 0.1%, frequency sweep range from 0.1 to 10 Hz, and 20 data points. All tests were performed in triplicate.

#### 2.2.11 Determination of Wheat Dough Moisture Distribution

Dough prepared according to the method in Section 2.2.6 was slightly modified based on the approach of Li, Wang, Jiang, et al. (2023) and Li, Wang, Wang, et al. (2023), and it was allowed to sit at 30°C for 30 min. Accurately weighed dough samples of 2.0 ± 0.01 g were wrapped in polytetrafluoroethylene tape and placed into the nuclear magnetic resonance analyzer (Shanghai Niumai) tube. The samples were then positioned at the center of a permanent magnetic field (with a field intensity of 0.5 T) and scanned using the Carr–Purcell–Meiboom–Gill (CPMG) pulse sequence to determine the spin–spin relaxation time *T*_2_. The parameters were set as follows: number of sampling points (TD) = 21,016; sampling frequency (SW) = 200.00 kHz; sampling interval time (TW) = 3000 ms; number of echoes = 1000; echo time = 0.1 ms; and number of accumulations (NS) = 16. After detection, the data were saved, and the *T*_2_ inversion program was used to obtain the relaxation time inversion spectrum of the dough samples. Each sample was measured three times, and the results were expressed as the mean value ± standard deviation.

#### 2.2.12 Determination of Wheat Dough Microstructure

To obtain frozen dough using the same method described in Section 2.2.7, and referencing the method of Li et al. (2019) with slight modifications, the frozen dough was cut into chunks and placed on a specimen holder with double‐sided adhesive. A gold coating was applied using ion sputtering, and the microstructure of the okara dough was observed and photographed at magnifications of 500× and 1000× under a scanning electron microscope (SEM; JEOL, Japan).

#### 2.2.13 Statistical Analysis

All experiments were replicated at least three times, and the results were expressed as mean ± standard deviation. The results were analyzed for variance (ANOVA) using IBM SPSS Statistics 26 (Chicago, USA) software. Duncan's test was used to compare the significant differences between samples. When *p* < 0.05, the differences between samples were considered statistically significant. Origin2022 software was used for all plotting and calculations.

## 3 Analysis of Results

### 3.1 Basic Composition of Okara

The elemental composition of pressed okara before and after extrusion treatment is shown in Table 1. After extrusion, the moisture, fat, SDF, IDF, and TDF contents of okara on a dry basis were 11.3%, 5.7%, 17.8%, 9.2%, and 51.3%, respectively, compared to the okara before extrusion. IDF decreased by 6%, SDF content increased by 6%, and TDF remained unchanged, indicating that the extrusion process caused cellulose and hemicellulose degradation in IDF (Nikinmaa et al. 2023). This is consistent with the results of previous studies, such as Wu et al. (2025), which found that extrusion can increase the content of SDF. The protein content of pressed okara remained almost unchanged. Still, it has been reported that extrusion can affect proteins' hydration capacity and denaturation, thereby influencing their biological activity or efficacy, such as increasing digestibility and reducing pancreatic protease inhibitor activity (Xie 2025). After extrusion, the fat content of okara decreased from 7.53% to 5.70%, which is consistent with the results of Wang et al. (2019). This may be due to the formation of fat‐protein complexes under high temperature and high pressure during the extrusion process, which prevents them from being extracted by petroleum ether. Meanwhile, the high temperature and high pressure increase the melting loss of lipids, as evidenced by the residual oil stains found on the screw (Kaur et al. 2022).

**TABLE 1:** Changes in basic composition of okara before and after extrusion.

Sample	Moisture/%	Fat/%	Protein/%	SDF/%	IDF/%	TDF/%	Ash/%
RO	11.42 ± 0.52^a^	7.53 ± 0.56^a^	17.60 ± 0.54^a^	2.85 ± 0.18^b^	57.30 ± 1.34^a^	60.75 ± 2.54^a^	4.05 ± 0.12^a^
EO	11.31 ± 0.49^a^	5.70 ± 0.27^b^	17.83 ± 0.61^a^	9.24 ± 0.36^a^	51.08 ± 1.78^b^	60.52 ± 2.83^a^	3.87 ± 0.09^a^

*Note:* Values with different letters (mean ± SD, *n* = 3) indicate significant differences (*p* < 0.05).

### 3.2 Analysis of Water‐ and Oil‐Holdinwell Well as Swelling Capacity of Okara

**FIGURE 1:**
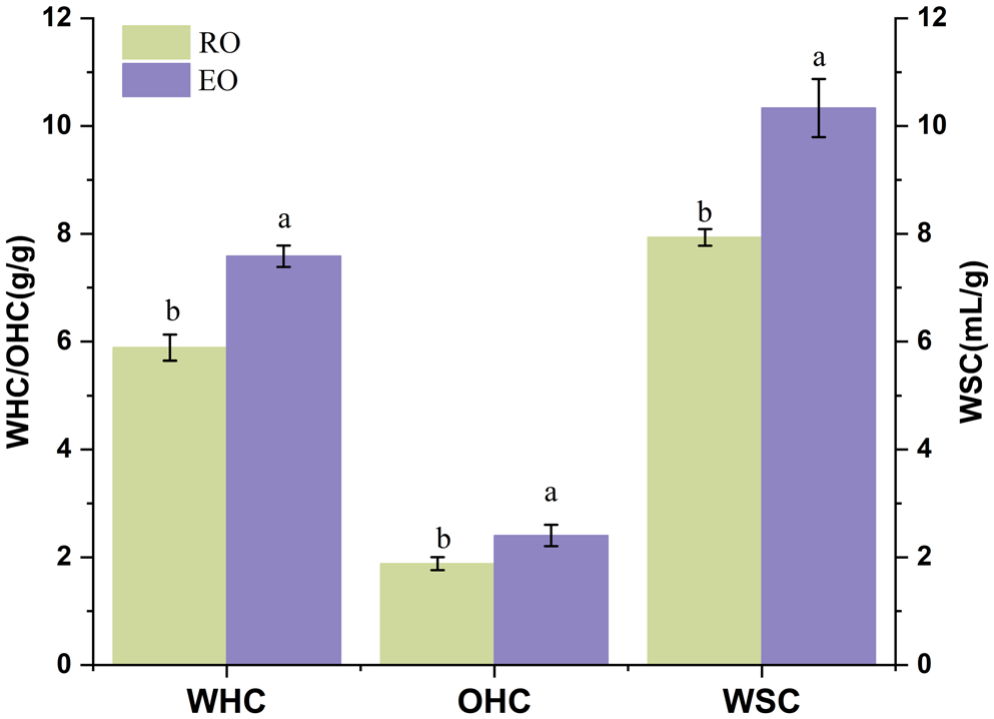
Effect of water and oil holding as well as the swelling capacity of okara before and after modification.

From Figure 1, it can be seen that the WHC, OHC, and WSC of okara after screw extrusion show an increasing trend. Among them, WHC increased from 5.89 (g/g) to 7.58 (g/g), and WSC rose from 7.93 (g/mL) to 10.33 (g/mL). The OHC increased from 1.88 (g/g) to 2.40 (g/g). This is because extrusion modification subjects okara to multiple factors such as high temperature, high pressure, and shear force, which make its internal structure looser, exposing more hydrophilic and hydrophobic groups. The exposure of hydrophilic groups is beneficial for improving the WHC and WSC, while the exposure of hydrophobic groups enhances the affinity of okara for oils, thereby increasing the OHC (Naumann et al. 2021). In addition, extrusion modification also increases the content of SDF in okara, which helps to improve the hydration properties of okara. The large number of hydrophilic groups in the molecular structure of SDF enables it to absorb more water than its weight, further enhancing the hydration capacity of okara (Lei et al. 2025). Moreover, the good hydration properties of SDF endow it with various beneficial functions in the intestine, such as increasing the viscosity of food, delaying or preventing the excessive absorption of cholesterol in food, and promoting intestinal peristalsis, thus reducing the risk of constipation and colorectal cancer (Wang et al. 2020).

### 3.3 Wheat Flour Pasting Characterization

**FIGURE 2:**
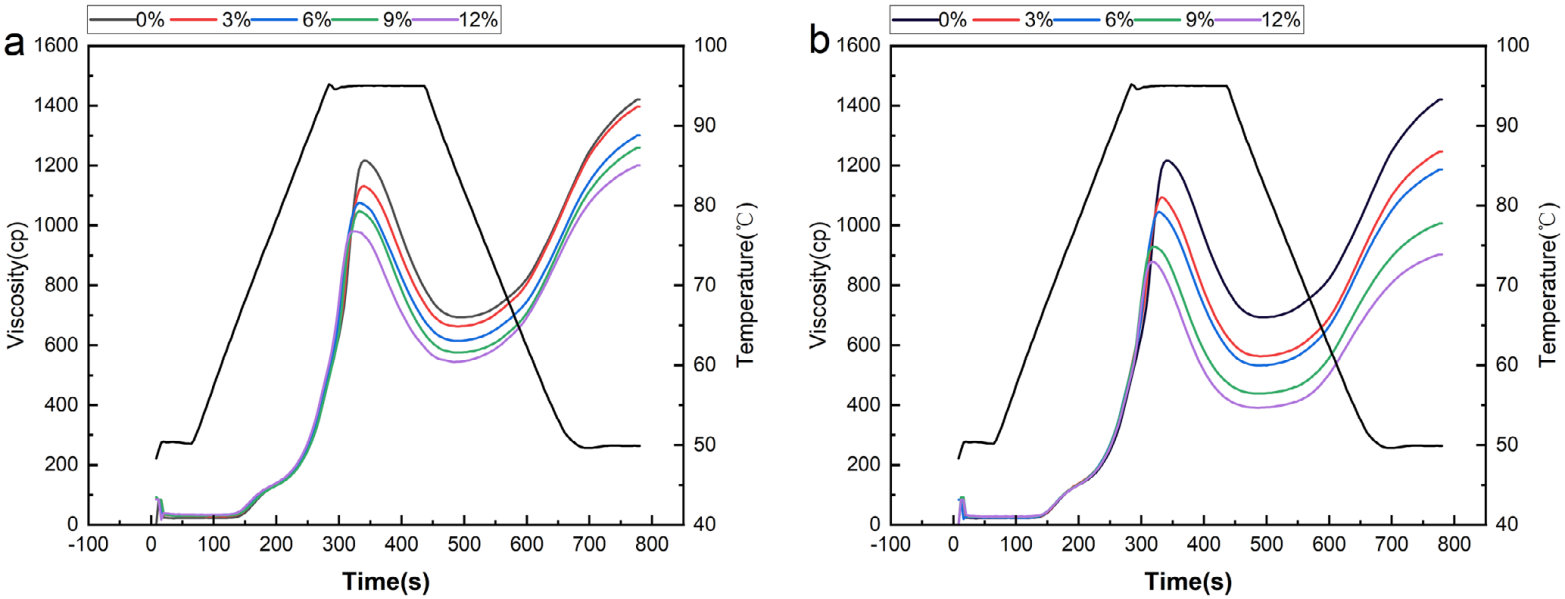
Gelatinization curve characteristics of mixed wheat flour: (a) denotes the raw material okara‐wheat mixed flour, (b) represents extruded okara‐wheat mixed flour (0%, 3%, 6%, 9%, and 12%) indicating different levels of okara added.

The pasting characteristics of wheat flours with different RO and EO contents are shown in Figure 2 and Table 2. During the RVA heating process, the viscosity of both sample groups initially increases rapidly with temperature until reaching a peak at 95°C. As the temperature is maintained at its peak, the viscosity gradually decreases. When the temperature decreases further, the viscosity decreases to a certain extent before progressively increasing again, possibly due to the dominance of wheat starch granules in the samples. Starch granules comprise a granular structure formed by hydrogen bonding of individual starch molecules. During heating, starch granules absorb water molecules and swell, increasing viscosity and reaching a peak. Further heating causes hydrogen bonds to break, starch granules to rupture, and a large amount of amylose to be released, resulting in viscosity reduction. Subsequent cooling leads to a decrease in temperature, the orderly alignment of amylose molecules, and further hydrogen bonding between chains, causing viscosity to rise again (Patil et al. 2016). During the subsequent cooling process, as the temperature decreases, the arrangement of amylose molecules tends to become more ordered, and more hydrogen bonds form between the chains, resulting in a rise in viscosity again.

**TABLE 2:** Pasting characteristics of wheat flour blends.

Sample	Okara addition/%	Peak visit/cp	Trough viscosity/cp	Breakdown/cp	Final visc/cp	Setback values/cp	Temperature/°C
RO	0%	1217.33 ± 6.51^a^	693.00 ± 19.01^a^	523.33 ± 12.90^a^	1421.00 ± 9.00^a^	727.00 ± 11.14^a^	87.33 ± 0.10^b^
	3%	1130.67 ± 7.02^b^	659.33 ± 8.51^b^	472.33 ± 12.22^bc^	1397.00 ± 8.66^b^	739.33 ± 19.14^a^	87.28 ± 0.25^b^
	6%	1073.00 ± 1.00^d^	614.00 ± 9.54^c^	461.67 ± 7.51^c^	1301.00 ± 5.29^c^	688.67 ± 12.50^b^	88.07 ± 0.16^a^
	9%	1050.33 ± 5.77^e^	574.33 ± 9.50^d^	472.33 ± 11.37^bc^	1260.33 ± 7.09^d^	683.67 ± 4.04^b^	87.187 ± 0.13^b^
	12%	979.67 ± 4.93^f^	544.33 ± 7.77^e^	435.67 ± 1.53^d^	1230.33 ± 10.12^f^	680.33 ± 11.15^b^	86.40 ± 0.18^c^
EO	3%	1094.00 ± 7.00^c^	562.67 ± 9.50^d^	531.33 ± 16.50^a^	1247.00 ± 5.00^e^	683.67 ± 3.51^b^	87.23 ± 0.10^b^
	6%	1045.67 ± 5.51^e^	537.33 ± 4.51^e^	513.33 ± 5.13^a^	1187.00 ± 6.56^g^	649.00 ± 6.25^c^	87.30 ± 0.25^b^
	9%	930.00 ± 5.00^g^	440.67 ± 9.02^f^	491.00 ± 12.00^b^	1007.00 ± 6.08^h^	568.00 ± 8.00^d^	87.25 ± 0.10^b^
	12%	878.67 ± 7.51^h^	387.67 ± 2.52^g^	489.33 ± 4.04^b^	903.00 ± 5.00^r^	513.00 ± 7.00^e^	87.15 ± 0.20^b^

*Note:* Values with different letters (mean ± SD, *n* = 3) indicate significant differences (*p* < 0.05).

Notably, the peak viscosity, trough viscosity, and final viscosity of the samples in the RO group were significantly higher than those in the EO group (*p* < 0.05). This can be primarily attributed to the presence of more intact raw starch granules in RO, which possess higher swelling capacity during gelatinization. Moreover, as the addition amount increased, the decreasing trend of viscosity was more significant in the EO group (Table 2). This might be because the particles in RO had relatively larger and non‐uniform shapes, resulting in poor dispersibility in the wheat flour system. These larger particles might form local aggregates during the gelatinization process, affecting the uniformity and fluidity of the entire system. After the extrusion treatment of okara, the particles became smaller and more uniform, and were more evenly dispersed. The specific surface area increased significantly, enabling them to compete more efficiently with starch for binding water molecules. Through hydrogen bonding and capillary action, EO particles formed a dense hydration layer on the surface of starch, hindering the swelling of starch and the dissolution of amylose, thus more significantly reducing the peak viscosity (Rachman et al. 2020). In addition, the content of SDF in EO was higher than that in RO. SDF contains a large number of hydrophilic groups, which can preferentially bind to water molecules, thereby reducing the amount of water available for starch and further reducing the viscosity of the system. This result is consistent with the findings of Zhou et al. (2021). The decrease in the setback value indicates an enhancement in the anti‐aging ability of starch. This may be because the increase in okara content causes SDF and amylose in the mixed flour to form complexes through hydrogen bonding, hindering their ordered recrystallization and leading to a decrease in the setback value (Boita et al. 2016).

### 3.4 Analysis of Wheat Flour Properties

The results measured by the farinograph are one of the important indicators for evaluating dough characteristics. The farinograph characteristics of wheat mixed flour are shown in Table 3. With the increasing amount of okara added, in addition to the rising trend of water absorption (WA), the values of all other parameters show a decreasing trend. The WA of EO is higher than that of RO. This may be because the surface of the extruded okara has more pores, which facilitates the penetration of water into it. In addition, the higher content of SDF in EO may contribute to this characteristic. This is consistent with the experimental results reported by Yoshida et al. (2023).

**TABLE 3:** Flour characteristics of okara wheat blends.

Sample	Okara addition/%	WA/%	DT/min	DS/min	ST/FU
RO	0	68.10 ± 0.56^f^	17.60 ± 0.36^a^	9.43 ± 0.009^bc^	53.00 ± 1.00^e^
	3	72.20 ± 0.76^e^	15.35 ± 0.55^b^	8.92 ± 0.61^cd^	66.40 ± 2.50^d^
	6	74.00 ± 0.50d	14.28 ± 1.02^c^	8.44 ± 0.42^d^	71.20 ± 2.64^c^
	9	77.83 ± 0.47^c^	12.65 ± 0.58^e^	6.61 ± 0.29^e^	79.30 ± 1.53^b^
	12	82.70 ± 0.46^b^	11.67 ± 0.71^e^	5.62 ± 0.15^f^	86.30 ± 2.08^a^
EO	3	73.93 ± 0.60^d^	16.79 ± 0.69^a^	11.65 ± 0.18^a^	35.30 ± 4.73^f^
	6	77.50 ± 0.70^c^	15.66 ± 0.29^b^	9.93 ± 0.25^b^	49.30 ± 1.72^e^
	9	82.90 ± 0.50^b^	14.90 ± 0.60^bc^	8.51 ± 0.40^d^	70.30 ± 1.20^cd^
	12	85.50 ± 0.80^a^	12.92 ± 0.31^d^	6.34 ± 0.34^e^	83.00 ± 0.97^ab^

*Note:* Values with different letters (mean ± SD, *n* = 3) indicate significant differences (*p* < 0.05).

The dough development time (DT) refers to the duration required for the interaction between water and flour until the dough is formed. The dough softening degree (ST) represents the ability of the dough, once formed from flour, to withstand mechanical mixing. The dough stability (DS), which is inversely proportional to the ST, is an important indicator for measuring the strength of gluten. These three indicators are closely related to dough quality and can intuitively reflect the effects of the amount of added okara and the modification of okara on dough quality (Meng et al. 2022). As the additional amount of okara increases, both the dough DT and the dough DS decrease, while the dough ST increases. The interaction between okara and flour intensifies. The DF in the okara interferes with the connection of gluten proteins and the formation of the gluten network, resulting in a shorter DT. The addition of okara disrupts the integrity and continuity of the gluten network. DF competes with gluten proteins for water and creates physical barriers during mixing, preventing the further cross‐linking and strengthening of gluten proteins, which leads to a lower DS and weakened gluten strength, manifested as an increase in ST. The dough with the addition of modified EO is superior to that with RO. This is because the SDF in EO has a high water‐holding capacity, which can make the water distribution in the dough more uniform, enable the gluten protein molecules to connect in an orderly manner, alleviate the interference of okara on gluten formation, and improve the DT. In terms of DS, SDF not only retains water but also promotes the formation of a macromolecular network structure by gluten proteins, enhancing the dough's resistance to mixing and its stability. Regarding ST, the gel‐like substance formed by SDF after absorbing water can fill the gaps in the gluten network, reducing damage during mixing, lowering the dough softening degree, and improving the dough quality (Villasante et al. 2022).

### 3.5 Analysis of Wheat Dough SH and SS

**FIGURE 3:**
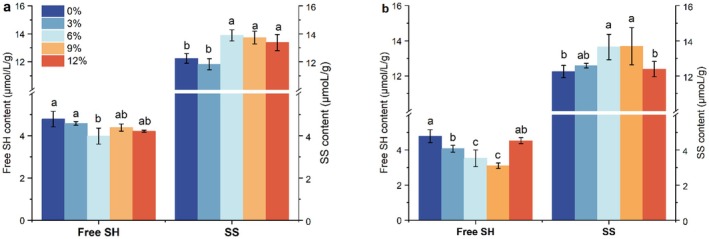
Effect of okara on SH_F_ and SS of dough (a, is adding ROD; b, for adding EOD). Dots (mean ± SD, *n* = 3) with different letters have mean values that are significantly different (*p* < 0.05).

SS are important chemical bonds that stabilize the gluten protein network structure and are widely present in the gluten network. They are formed by the oxidation of two SHF groups in gluten proteins. A higher SS content in the protein network structure is more conducive to stability (Shu et al. 2022). As shown in Figure 3, the changes in SHF and SS contents in ROD and EOD with different addition amounts can be observed. With the increase in okara addition, the SHF content in both doughs showed a trend of decreasing and then increasing, while SS content showed a trend of increasing and then decreasing. Compared with ROD, the overall SHF content in EOD with added okara was lower, while the SS content remained relatively consistent. This may be because the extrusion process generates high temperature and pressure, destroying stable secondary structure hydrogen bonds and exposing the hydrophobic and thiol groups inside the okara protein chain. Intermolecular SS and hydrophobic interactions promote protein aggregation and cross‐linking, thereby enhancing protein aggregation through hydrophobic contact (Liu et al. 2022). When the addition level of ROD is 6% and that of EOD is 9%, the dough has the highest SS content and the lowest SHF content, which is significantly different from the control group (*p* < 0.05), indicating that the addition of okara promotes the transformation of SHF into SS in gluten protein molecules, with a more pronounced effect in EOD. When the amount of okara is too small or too large, the SHF content in the dough increases and the SS content decreases. This may be because the proteins in okara compete with the proteins in flour for binding sites, thereby inhibiting the formation of SS (Lu et al. 2023).

### 3.6 Secondary Structure Analysis of Wheat Dough Proteins

**FIGURE 4:**
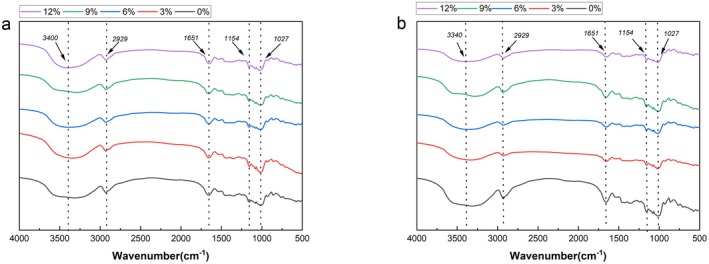
FTIR spectra of gluten proteins. (a) represents ROD, (b) represents EOD, and (0%, 3%, 6%, 9%, and 12%) indicates different levels of okara addition.

FTIR is a commonly used method for analyzing the secondary structure of macromolecules. Different functional groups exhibit characteristic frequencies in the infrared spectrum, which can be used to preliminarily determine whether RO and EO impact gluten proteins. As shown in Figure 4, wheat dough exhibits multiple characteristic absorption peaks in the 4000–500 cm^−1^ range. The spectra of ROD and EOD at different addition levels are similar, and no new infrared absorption peaks appear or disappear. In the 3200–3700 cm^−1^ range, the absorption peaks represent the stretching vibration of −OH, while at 2929 cm^−1^, they represent the stretching vibration peak of C‐H (Zhou et al. 2019). The absorption peak at 1651 cm^−1^ represents the stretching vibration of the amide I band (C=O). Additionally, the range of 800–1200 cm^−1^ is considered the characteristic spectrum of starch, reflecting the stretching vibration peaks of starch C‐C, C‐OH, and C‐H bonds (Liu et al. 2022).

The different vibrational modes of C=O, C‐N, and N‐H in proteins are related to their secondary structure. The amide I band (mainly the stretching vibration of C=O), 1600–1700 cm^−1^ contains information about β‐turns, α‐helices, random coils, and β‐sheets (Liu et al. 2022). Among them, α‐helices and β‐sheets play a crucial role in stabilizing the dough structure; α‐helices are mainly supported by hydrogen bonds in their helical structure, while β‐sheets form a layered structure under the action of hydrogen bonds. Generally, the higher the content of these structures, the denser the gluten network formation and the better the viscoelasticity (Hu et al. 2017). Conversely, higher contents of β‐turns and random coils lead to poorer gluten network structural properties because they represent disordered structures (Lou et al. 2023). Therefore, we analyze the secondary structure of gluten proteins in dough by calculating the peak areas of different structures.

**FIGURE 5:**
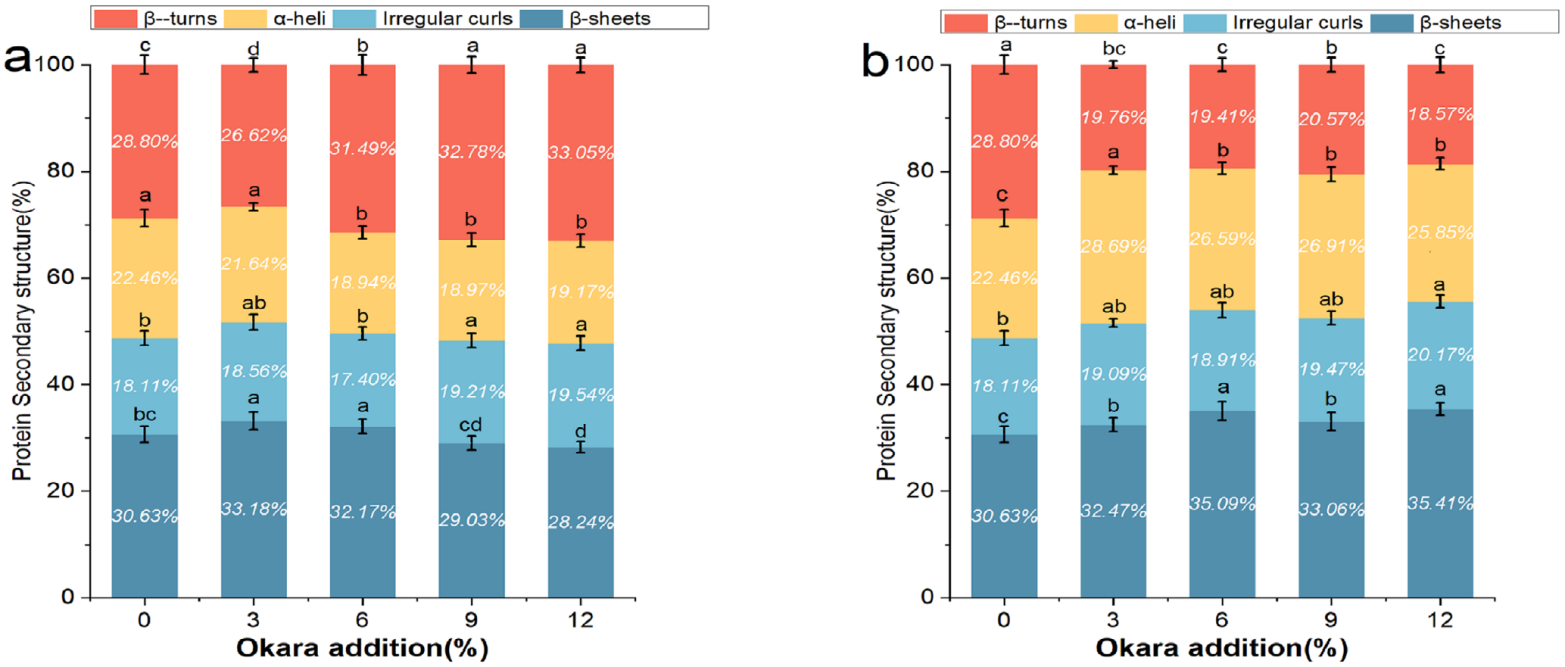
Protein secondary structure content in the dough. (a) Represents ROD and (b) Represents EOD. Dots (mean ± SD, *n* = 3) with different letters have mean values that are significantly different (*p* < 0.05).

From Figure 5, we can see that in ROD, β‐sheet and β‐turn are the main components of the protein secondary structure, accounting for 28.24%–32.18% and 26.62%–32.78%, respectively. α‐helixes and irregular curls account for 18.94%–22.46% and 17.40%–19.54%, respectively. Compared with the control group, as the additional amount of okara increases, the content of β‐turn in ROD shows an upward trend, while the content of β‐sheet tends to decline. This may be because the increase in the additional amount of okara leads to a decrease in gluten content, thereby reducing the dough's stability. This is consistent with the results of Li et al. (2024) In EOD, however, α‐helix and β‐sheet are the main components of the protein secondary structure in the dough. With the increase of the okara addition amount, their contents show a trend of increasing first and then decreasing, accounting for 25.85%–28.69% and 32.47%–35.41%, respectively. The increase in α‐helix makes the dough more orderly (Liu et al. 2016). Compared with the dough with unextruded okara, adding EOD makes the gluten structure more inclined to the β‐sheet conformation. This may be because the high water absorption capacity of okara DF destroys the hydrogen bonds that maintain the β—turn structure, thus promoting the formation of small molecules. Then, these small molecules aggregate through non‐covalent interactions, leading to an increase in the proportion of β—sheets. On the other hand, the competitive water absorption between okara DF and gluten proteins results in the dehydration of gluten proteins, altering their conformation and redistributing moisture, leading to gluten dehydration. This structural transformation promotes protein aggregation, thus forming a tightly interconnected three‐dimensional viscoelastic network structure in the dough (Li et al. 2024).

### 3.7 Analysis of Wheat Dough Texture

Research indicates that the texture of dough is significantly correlated with the production of final products and is also an important indicator for evaluating dough‐based products. As shown in Table 4, the influence patterns of different addition amounts of RO and EO on the dough are basically the same. For the okara dough, as the addition amount increases, the hardness and chewiness show an upward trend, and this trend is more obvious in the ROD group (reaching 1222.12 g at addition of 12%), while the increase in the EOD group is relatively gentle (1058.26 g at 12% addition). This may be because the proportion of SDF increases after extrusion modification. The hydrophilic groups of SDF competitively bind water through hydrogen bonds, reducing the competition for water by IDF (Jia et al. 2019).

**TABLE 4:** Textural analysis of wheat dough.

Sample	Okara addition/%	Hardness/g	Springiness	Cohesiveness	Chewiness/mJ
ROD	0	648.46 ± 32.48^d^	0.709 ± 0.02^abcd^	0.740 ± 0.02^d^	344.06 ± 45.17^fg^
	3	715.58 ± 13.30^d^	0.616 ± 0.02^e^	0.720 ± 0.02d^e^	318.62 ± 40.05^fg^
	6	849.35 ± 3.25^c^	0.660 ± 0.13^cde^	0.800 ± 0.02b^c^	468.33 ± 11.88^de^
	9	1070.26 ± 75.81^b^	0.652 ± 0.01^de^	0.830 ± 0.03^ab^	588.75 ± 75.73^bc^
	12	1222.27 ± 60.18^a^	0.696 ± 0.05^bcd^	0.870 ± 0.01^a^	762.67 ± 55.17^a^
EOD	3	652.85 ± 26.50^d^	0.715 ± 0.02^abc^	0.680 ± 0.03^e^	315.72 ± 68.79^g^
	6	735.32 ± 57.54^d^	0.766 ± 0.04^a^	0.730 ± 0.02^d^	411.53 ± 23.33^ef^
	9	885.46 ± 13.75^e^	0.719 ± 0.03^abc^	0.770 ± 0.03^cd^	543.71 ± 18.61^cd^
	12	1058.26 ± 65.10^b^	0.741 ± 0.05^ab^	0.8200 ± 0.03^ab^	655.37 ± 26.15^b^

*Note:* Values with different letters (mean ± SD, *n* = 3) indicate significant differences (*p* < 0.05).

The three‐dimensional colloidal network formed by SDF can bind to gluten proteins through hydrogen bonding to form a “fiber–protein” structure, thereby alleviating and reducing the hardness of the dough, which is consistent with the experimental results of Wang et al. (2023). In terms of elasticity, the overall elasticity of EOD is better than that of ROD. The elasticity of EOD reaches the highest value (0.766) at an addition of 6%, which is significantly higher than that of ROD (*p* < 0.05). This may be because EOD contains more SDF, and SDF shows strong viscosity after absorbing water, enhancing the interaction between water, starch, and gluten, thus improving the overall quality of the wheat dough (Sha et al. 2023). From the above conclusions, it can be seen that adding extruded okara to the dough balances the negative impact of fiber addition on texture, which is closely related to the ratio of SDF to IDF in the okara. Moreover, the addition of EO is optimal for maintaining the dough's elasticity and controlling the increase in hardness.

### 3.8 Analysis of Dynamic Rheological Properties of Wheat Doughs

The rheological properties of the dough reflect the changes in the viscoelasticity of the polymer network in the dough after okara is kneaded with it. The elastic modulus (G′), also known as the storage modulus, characterizes the elastic properties of the dough, corresponding to its elasticity; the viscous modulus (G″), also known as the loss modulus, characterizes the viscous properties of the dough, corresponding to its flowability and viscosity. The loss tangent (tan δ) is the ratio of viscosity to elasticity measured in the dough, i.e., tanδ = G″/G′. When tanδ is lower, it indicates a higher proportion of elasticity in the dough, weaker flowability, and a higher proportion or greater polymerization degree of high polymers in the dough; conversely, when tanδ is higher, it indicates a higher proportion of viscosity in the dough, better flowability, and a higher proportion of low‐polymerized components (Lu et al. 2023).

**FIGURE 6:**
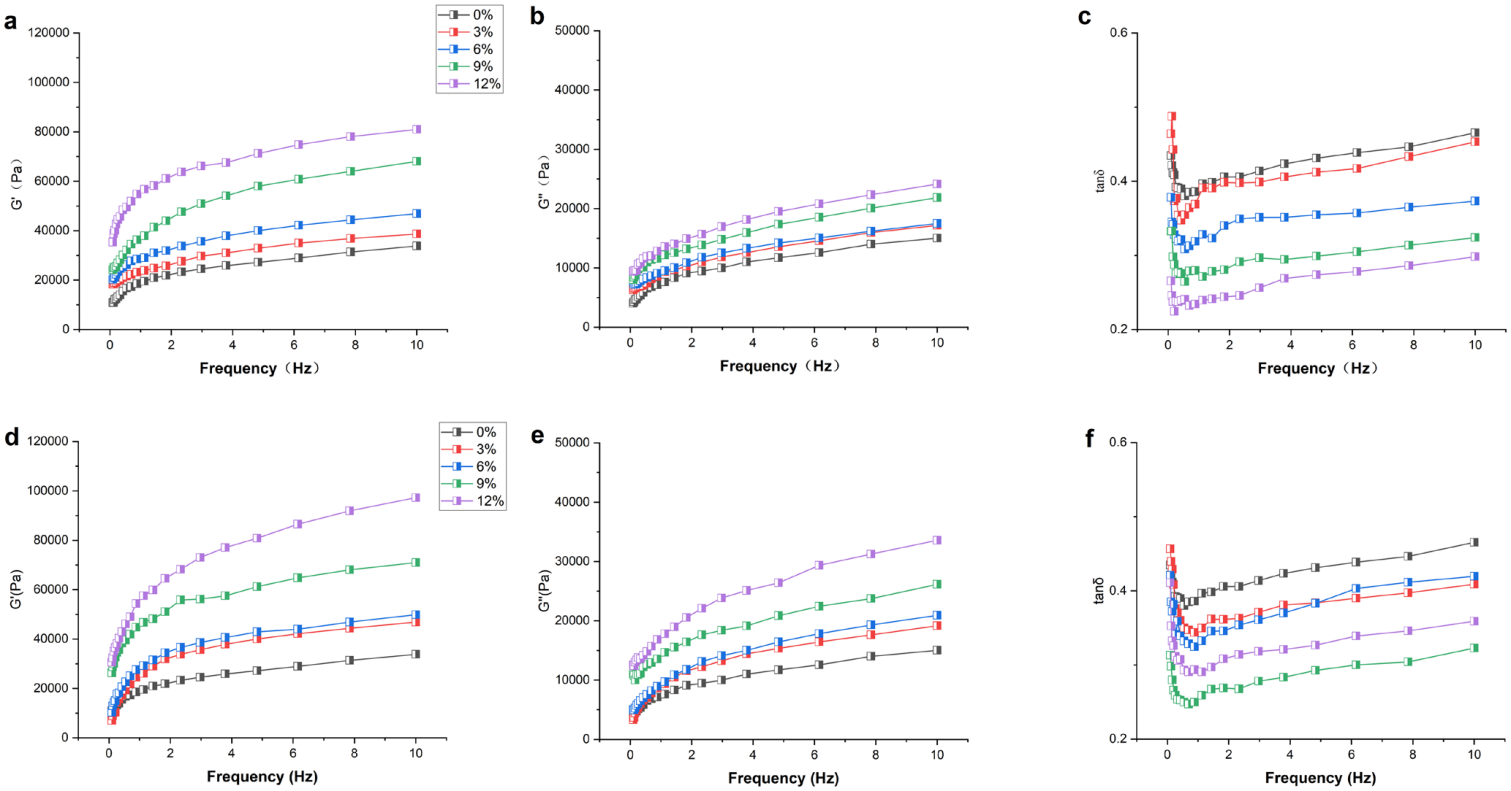
Dough dynamic rheology, (a–c) for ROD and (d–f) EOD, (0%, 3%, 6%, 9%, and 12%) representing different okara additions.

Figure 6 depicts the variations in G′, G″, and tanδ of ROD and EOD under different oscillation frequencies. It is clearly observable from the figure that for all dough samples, G″ is smaller than G′, and the values of tanδ are all less than 1. This phenomenon fully indicates that the viscoelastic behavior of these doughs exhibits solid‐like viscoelastic behavior. That is, when the dough is subjected to force, it has a certain elastic recovery ability while also experiencing a certain degree of viscous deformation, presenting overall characteristics similar to those of a solid (Zhang et al. 2024). Upon further analysis, it is found that the storage G′ and G″ of all dough samples increase with the increase in the additional amount of okara. This result is consistent with the findings of Liu et al. (2019). Notably, the modulus values of the EOD group are higher than those of the ROD group. This difference suggests that the dough with added extruded okara has higher elasticity than the dough with added RO. This phenomenon is closely related to the additional amount of okara. Okara can play an important structural connection role in the dough system. It can connect some macromolecular chains in the dough, especially wheat gluten, and promote the polymerization reaction between proteins. Through this polymerization, a firm and ordered network structure is formed in the dough, making the dough present a solid‐like state with reduced fluidity, and thus significantly increasing the elasticity of the dough (Mei et al. 2024; Sui et al. 2018).

### 3.9 Analysis of Wheat Dough Moisture Distribution

**FIGURE 7:**
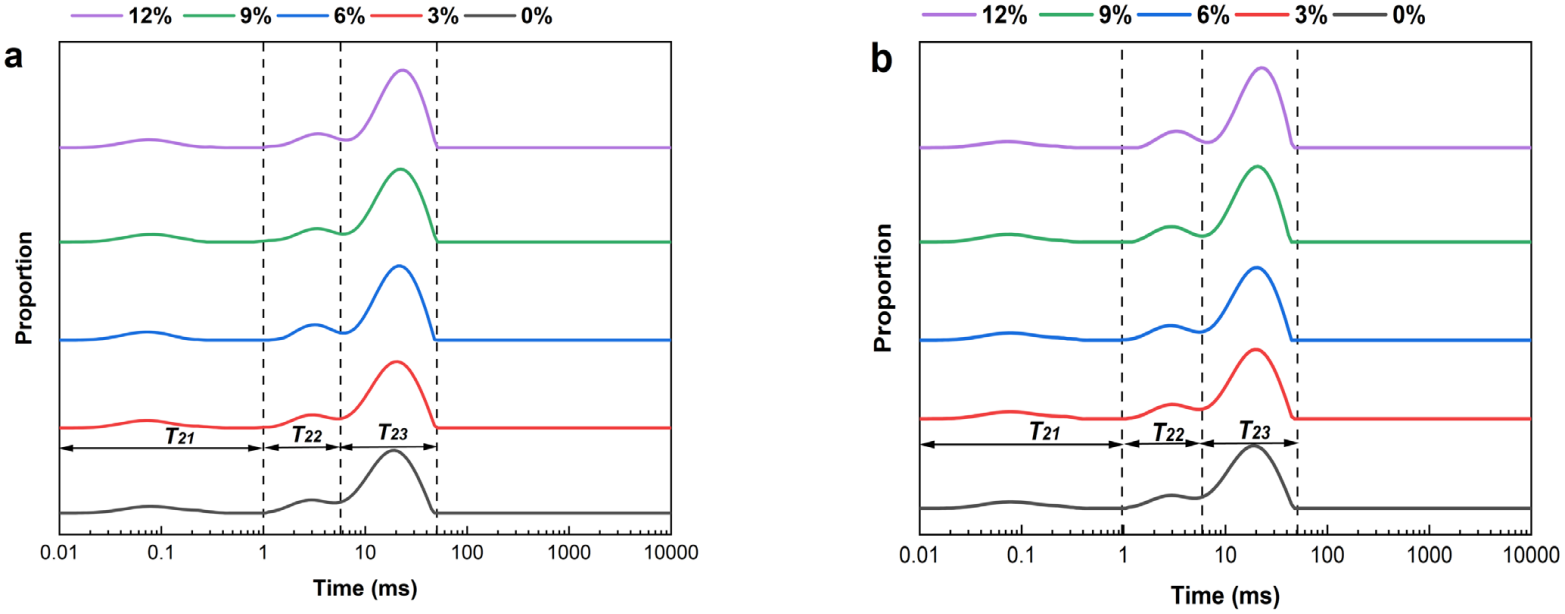
*T*_2_ relaxation maps of dough moisture distribution. (a) Represents ROD, (b) represents EOD. (0%, 3%, 6%, 9%, and 12%) denote different levels of okara addition.

Water is an essential component of food, playing a crucial role, particularly in processing dough‐based products (Yue et al. 2023). To investigate the strength of the interaction between wheat flour and different levels of okara before and after extrusion, as well as the types of water present in the dough, this experiment used Low field NMR (LF‐NMR) technology to measure the *T*_2_ relaxation time of the dough (Table 5, Figure 7). The *T*_2_ relaxation time of dough is associated with the binding strength and mobility of hydrogen protons. A shorter *T*_2_ relaxation time indicates a higher degree of binding or lower mobility of hydrogen protons, whereas a longer *T*_2_ relaxation time suggests weaker binding or higher mobility of hydrogen protons (Liu et al. 2016). We detected three proton populations (*T*_21_, *T*_22_, and *T*_23_) using LF‐NMR testing CPMG. Based on the relaxation times, *T*_21_ (0.01–1 ms) is categorized as tightly bound water, while *T*_22_ (1–6 ms) is categorized as loosely bound water, and *T*_23_ (6–40 ms) for water that does not flow easily. The y‐axis represents the signal amplitude of protons, where the peak area of *T*_2_ (*A*_21_, *A*_22_, and *A*_23_) represents the relative content of hydrogen protons and the water absorption capacity of hydrophobic components. Tightly bound water refers to molecules bound to gluten proteins through hydrogen bonding, such as amino, carboxyl, and hydroxyl groups, with low mobility, hence termed tightly bound water. Loosely bound water exhibits weaker binding than tightly bound water but stronger than free water, thus termed loosely bound water. In this experiment, free water was not observed. This phenomenon may be attributed to the dough preparation process, where the dough undergoes extensive fermentation, allowing gluten proteins, starch, or fibrous materials to sufficiently bind with water, forming tightly and loosely bound water. Consequently, the content of free water is significantly reduced (Lu et al. 2022).

**TABLE 5:** *T*_2_ relaxation time and peak area ratio of wheat doughs.

Sample	Okara addition/%	*T*_21_/ms	*T*_22_/ms	*T*_23_/ms	*A*_21_/%	*A*_22_/%	*A*_23_/%
ROD	0	0.08 ± 0.04^a^	2.9 ± 0.15^d^	19.34 ± 1.01^b^	9.46 ± 0.21^a^	15.76 ± 0.78^bc^	74.81 ± 2.15^a^
	3	0.07 ± 0.00^a^	2.97 ± 0.10^cd^	20.73 ± 1.99^ab^	9.31 ± 0.17^a^	15.36 ± 0.39^bc^	75.30 ± 3.10^a^
	6	0.08 ± 0.00^a^	3.18 ± 0.11^bc^	22.22 ± 2.00^ab^	9.27 ± 0.22^a^	16.28 ± 0.63^bc^	74.50 ± 1.95^a^
	9	0.08 ± 0.00^a^	3.41 ± 0.10^ab^	22.15 ± 2.91^ab^	9.51 ± 0.27^a^	14.88 ± 0.13^c^	75.63 ± 2.03^a^
	12	0.08 ± 0.00^a^	3.41 ± 0.19^ab^	23.817 ± 2.01^a^	9.38 ± 0.18^a^	15.02 ± 0.47^bc^	75.65 ± 2.60^a^
EOD	3	0.07 ± 0.01^a^	2.97 ± 0.00^cd^	19.34 ± 1.56^b^	9.34 ± 0.20^a^	16.01 ± 0.45^abc^	74.08 ± 3.06^a^
	6	0.08 ± 0.01^a^	2.93 ± 0.15^d^	20.729 ± 2.03^ab^	9.32 ± 0.31^a^	16.31 ± 0.95^abc^	74.39 ± 3.51^a^
	9	0.08 ± 0.00^a^	2.97 ± 0.00^cd^	20.398 ± 1.65^ab^	9.54 ± 0.25^a^	16.41 ± 1.16^ab^	73.90 ± 3.40^a^
	12	0.08 ± 0.01^a^	3.42 ± 0.18^a^	22.549 ± 2.41^ab^	7.30 ± 0.30^b^	17.57 ± 1.07^a^	74.90 ± 4.51^a^

*Note:* Values with different letters (mean ± SD, *n* = 3) indicate significant differences (*p* < 0.05).

From Table 5, it can be seen that *A*_23_ accounts for more than 70%, indicating that the moisture in the dough mainly exists in a form that is not easily flowable. Compared with ROD, the relaxation time of EOD is shorter. *T*_22_ and *T*_23_ decrease relatively, and *A*_23_ decreases significantly, thereby reducing the flowability of water in the dough. This may be because EOD has a higher content of SDF, which has a higher water‐holding capacity, and with the increase of okara content, there are more hydrogen bonding sites in the DF. This can form a complex system with proteins and starches in the dough through hydrogen bonding, hydrophobic interactions, etc., collectively trapping water molecules, increasing the interaction between water molecules and starches, proteins, etc., leading to tighter binding of water in the dough, thereby reducing its flow ability (Li, Wang, Jiang, et al. 2023; Li, Wang, Wang, et al. 2023).

### 3.10 Analysis of Wheat Dough Microstructure

**FIGURE 8:**
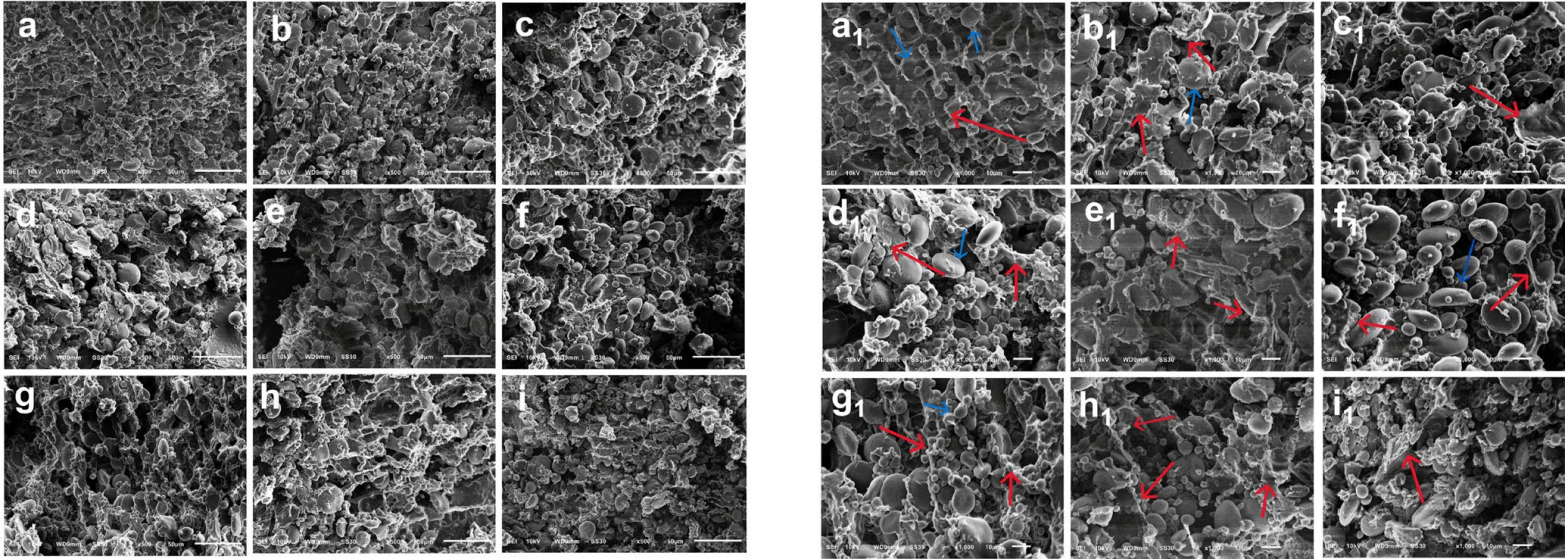
SEM images of dough, where (a–e) represent ROD with addition levels of 0%–12%. (f–i) Represent EOD with 3%–12% addition levels. Blue arrows indicate starch granules; red arrows denote the gluten network structure.

SEM has become an essential instrument for observing the microstructure of wheat dough due to its excellent resolution and ability to visualize three‐dimensional structures (Shen et al. 2021). Through Figure 8, we can observe the detailed microstructure of wheat dough. First, in the wheat dough without added okara (Figure 8a), we can see that the starch granules (indicated by the blue arrows) are evenly distributed around the large starch granules. They are tightly wrapped in the network structure formed by gluten proteins, presenting an ordered gluten structure. As the amount of RO added gradually increases (Figure 8b–e), the gluten structure of the wheat dough begins to break (in‐dicated by the red arrows). The starch granules begin to be exposed on the surface of the gluten protein network, resulting in uneven size distribution and a looser gluten network structure. In contrast, wheat dough with EO added (Figure 8f–i) exhibits similar issues. However, unlike unmodified okara dough, its surface is more uniform, and the gluten network structure is more ordered. Especially, the structure of EO dough containing 6% okara is more uniform than that of the other groups. This improvement may be attributed to the cross‐linking between SDF and gluten proteins through hydroxyl groups, hydrogen bonding, and hydrophobic interactions, which enhances the structural stability and viscoelasticity of the dough, thus making the network structure more compact. This is consistent with the results of the SS experiment (Jiang et al. 2023). When the amount of EO added is excessive, the surplus okara competes for water, affecting gluten formation and resulting in a loose network structure in the dough. Additionally, although EO has a higher content of SDF, it still contains a significant amount of IDF, which affects moisture distribution in the dough, leading to dehydration of some gluten proteins, thereby altering the gluten protein network structure and even causing partial collapse (Li, Wang, Jiang, et al. 2023; Li, Wang, Wang, et al. 2023). Overall, the microstructure of the mixed wheat dough shows that a low addition amount (≤ 9%) of okara helps to fill the network structure and improve the texture of the dough, while an excessive amount of okara hinders the formation of the gluten network and reduces dough quality.

## 4 Conclusions

This study found that after extrusion treatment, the IDF in soybean dregs was converted into SDF, significantly increasing the SDF content in soybean dregs. Compared with the addition of RO, when the addition ratio of EO was less than 12%, the positive effects on the dough were more significant. This finding is consistent with previous research results in this field. Specifically, the addition of EO could improve the water absorption capacity of the dough and increase the content of di‐sulfide bonds. Meanwhile, it prolonged the dough stability time and reduced dough hardness, free sulfhydryl content, viscosity, and retrogradation value. In addition, as the amount of soybean dregs increased, the dynamic G′ and G″ of the dough also showed an upward trend, while the tan δ value decreased. LF‐NMR results revealed the formation of two distinct water phases within the dough, with higher SDF content in EOD, thereby enhancing water‐binding capacity and increasing the proportion of tightly bound water in the dough. These findings suggest that interactions between okara and gluten influence the secondary structure of proteins. Particularly in EOD, the increase in α‐helix content and transformation of β‐turns to β‐sheets indicate partial dehydration of gluten, forming a more stable and aggregated gluten network. However, at a high addition level (12%), the gluten network structure of the dough was damaged, which was unfavorable for the subsequent production and processing of flour products. In conclusion, adding less than 12% EO to the dough helps to strengthen the gluten network structure, increase the SDF content, optimize the composition of the protein secondary structure, and enhance the dough's elasticity. This study provides a solid theoretical basis for the subsequent production and processing of flour products. In the future, further research can be conducted to explore how DF, proteins, and lipids in soybean dregs separately affect the rheological properties of the dough, so as to fully analyze the potential mechanisms of soybean dreg‐induced changes in the dough.

## Author Contributions

**Fengchen Zhou:** conceptualization (equal), investigation (equal), methodology (equal), resources (equal), writing – review and editing (equal). **Yangyang Cui:** conceptualization (equal), investigation (equal), methodology (equal), writing – original draft (supporting). **Shiming Wang:** data curation (equal), software (equal). **Xianjun Zou:** validation (equal). **Zhilong Chen:** validation (equal). **Yakun Shan:** formal analysis (equal). **Yu Zhang:** formal analysis (equal). **Sheng Li:** data curation (equal), funding acquisition (equal).

## Conflicts of Interest

The authors declare no conflicts of interest.

## Data Availability

All the data is in the article.
